# Longitudinal evaluation of Ocimum and other plants effects on the feeding behavioral response of mosquitoes (Diptera: Culicidae) in the field in Tanzania

**DOI:** 10.1186/1756-3305-1-42

**Published:** 2008-10-22

**Authors:** Eliningaya J Kweka, Franklin W Mosha, Asanterabi Lowassa, Aneth M Mahande, Michael J Mahande, Charles P Massenga, Filemoni Tenu, Ester E Lyatuu, Michael A Mboya, Emmanuel A Temu

**Affiliations:** 1 Tropical Pesticides Research Institute, Division of Livestock and Human Disease Vector Control, P.O. Box 3024, Arusha-Tanzania; 2 KCM College of Tumaini University P.O. Box 2240, Moshi, Tanzania; 3 Tanzania Wildlife Research Institute, P.O. Box 661, Arusha, Tanzania; 4 National Institute for Medical Research, Ubwari Field station, P.O.Box 81, Muheza, Tanzania; 5 Institute of Tropical Medicine, Nagasaki University, 1-12-4 Sakamoto, Nagasaki, Japan

## Abstract

**Background:**

The use of repellent materials from plants against nuisance insects is common with great potential to compliment existing malaria control programmes and this requires evaluation in the field. Ocimum plant species, *Ocimum suave *(Willd) and *O. kilimandscharicum *(Guerke) materials and their essential oils extracted by steam distillation were evaluated in the field and experimental huts for repellence, exophily and feeding inhibition effects against three mosquito species, *Anopheles arabiensis *(Patton), *An. gambiae *ss (Giles) and *Culex quinquefasciatus *(Say). The protective effect of essential oils from Ocimum plants were compared with N, N-diethly-3- methylbenzamide (DEET), a standard synthetic repellent. Also, the protective effect of fumigation by burning of repellent plants; *Ocimum suave, Ocimum kilimandscharicum*, *Azadirachta indica*, *Eucalyptus globules *and *Lantana camara *were tested in experimental huts and selected local houses.

**Results:**

In the field, protection by Ocimum plants from mosquito bites was high and there was small variation among different mosquito species. Protection efficiency was 93.4%, 91.98% and 89.75% for *An. arabiensis *while for *Cx*. *quinquefaciatus *it was 91.30%, 88.65% and 90.50% for DEET, *Ocimum suave *and *O. kilimandscharicum *respectively. In the experimental hut, deterrence induced by burning of Ocimum and other plants ranged from 73.1.0% to 81.9% for *An. arabiensis *and 56.5% to 67.8% for *Cx. quinquefaciatus*, while feeding inhibition was 61.1% to 100% for *An. arabiensis *and 50% to 100% for *Cx. quinquefaciatus*. Evaluations under field conditions confirmed high protective efficacy, enhanced feeding inhibition and house entry inhibition (Deterrence).

**Conclusion:**

This study shows the potential of *Ocimum suave and Ocimum kilimandscharicum *crude extracts and whole plants of *Ocimum suave, Ocimum kilimandscharicum*, *Azadirachta indica*, *Eucalyptus globules and Lantana camara *for use in protecting against human biting while the burning of plants reduces significantly the indoor resting mosquitoes.

## Background

Since ancient times, several plants and plant products have been used locally to repel or kill mosquitoes. There are several plants in sub-Saharan Africa reported to constitute effective repellents effect against arthropods of vector- borne disease [1-5]. Some of these plants, for example citronella and pyrethrum, have been commercialised and are effectively used as mosquito repellents [3].

In lower Moshi villages, we investigated whether whole plant and plant products derived from local areas can be used in combination with the bed nets to provide protection against malaria vectors and nuisance biting insects. Before starting such an investigation, we conducted an ethnobotanical survey to understand the common knowledge, attitude and practices, of local people, on the use of plant products for protection against mosquitoes and other biting insects. At Lower Moshi, *Ocimum suave, Ocimum kilimandscharicum*, *Azadirachta indica*, *Eucalyptus globules and Lantana camara *plants are common and known to have provide protection against mosquitoes [6]. These aromatic plants, *Ocimum suave *(OS) *and Ocimum kilimandscharicum *(OK) locally know as a broom "Ufagio", in the Kiswahili language, belong to the family Lamiaceae and are the focus of this study. Several plants of this family have been proven to have insecticidal and repellent effects, used widely against blood- feeding arthropods and those feeding on crops [7-11].

Although, treated mosquito nets have been proved to be effective in reducing child morbidity and mortality [1,12,13], there are still operational problems slowing down the scaling up of Insecticides Treated bed nets (ITN) usage such as seasonal variation of ITN use in the community, equity and access constraints, low rates of net re-treatment with insecticides and reports of insecticide resistance in malaria mosquitoes. With such problems facing the existing control measures against vector- borne diseases, there is a need to look for alternative and supplementary means to support existing control measures. Alternative, cost- effective and environmentally friendly bio-products such as plant repellents can potentially be improved to supplement existing vector- control measures [11]. Although there are many plant species used traditionally for protection against blood- feeding insects, there are few studies to illustrate their protective efficacy and or contribution to disease control. Following a survey conducted, OS and OK were the most common plants used as insects repellent by local communities at lower Moshi, north-eastern Tanzania [6]. This study evaluates deterrence, exophily and feeding inhibition effects of *Ocimum suave *(OS), *Ocimum kilimandscharicum *(OK), *Azadirachta indica *(AI) *Eucalyptus globules *(EG) and *Lantana camara *(LC) on three mosquito species, *Anopheles arabiensis *(Patton), *An. gambiae *ss (Giles) and *Cx. quinquefasciatus *(Say) in the field and experimental huts.

## Methods

### Study area

The surveys were taken from January to March 2006, at Lower Moshi villages (37°20' E, 3°21' S; 750 M above sea level), located 19 km South of Moshi town, on the foot slopes of Mount Kilimanjaro. The area is fully described elsewhere [6]. The study was conducted at Mabogini, Rau Kati, Chekereni and Mtakuja villages located at Mabogini ward with an estimated population of 20,614, 4871 households and average of 4.2 people per house [14].

### The protective efficacy of plant materials in experimental huts and village houses

The plant materials of OS, OK, AI, EG and LC, were the first five common plants used as repellents mentioned by the community members in previous study in this area [6]. The method used for insect protection is mainly burning of dry plant material. In this test, one kilogram of each plant material was burnt between 7 and 10 pm in selected houses in the community and experimental huts as commonly used in the community.

In the experimental hut trials, two huts were selected, the test hut and control hut. The experiment had a binary setting. One hundred (100) female mosquitoes of 3 to 6 days old of the same species were released in each hut with a person sleeping under an untreated bed net. The plant materials, particularly the leaves [6] were picked up within the community areas then dried in the sun for a day before use in this experiment.

Plant materials were burnt only in the test hut. The next day mosquitoes were collected in Window traps and verandahs. Physiological conditions (unfed, blood fed and gravid) of mosquitoes collected were observed, then provided with 10% sugar solution for 24 hrs to score mortality.

In the village trial, eight houses were selected and grouped into four pairs each with two houses, i.e. the control and experimental houses. Volunteers slept under an untreated net in each of the trial houses, to protect them from being exposed to wild mosquitoes that might be infected. Effects of plant repellents on mosquitoes were observed for four consecutive days.

### Field trial of extracts of Ocimum plants (community study)

Four houses and four pairs of volunteers performing man-landing catch (MLC) at each house were involved to evaluate the protective effect of essential oil extracts from OS and OK. The volunteers were provided with anti-malarial prophylaxis during the study period. The first group was treated with OK (a solution with 20% OK essential oil); second group treated with DEET at similar concentration (20%), third group with OS (a solution with 20% OS essential oil) and the fourth, a control group treated with a mixture of glycerine and acetone. The 20% solution of OK, DEET and OS were prepared by dissolving crude essential oil into glycerine and acetone to a final concentration of 20%. The proportion of major active ingredients in that sample (OK, OS and DEET) was used to derive the concentration of 20% which was used in this evaluation. The amount of oil used by volunteer was determined by measuring the weight of oil with bottle before and after application on feet. The repellent and control were applied on feet below the knee. Volunteers seating on chair 5 meters apart outside the house collected mosquitoes landing on their lower legs and on their feet using an aspirator [15]. Collected mosquitoes were grouped in hourly intervals and identified using a morphological key [16,17]. Experiments were 4 by 4 Latin square arrangement, for four days per week for sixty-four weeks. The exercises started at 18:00 h and ended at 22:00 h. Both insect collectors and treatments were interchanged to prevent bias. Experiments were done for 4 days in a week and each treatment was rotated with same pair of volunteers. Treatments were interchanged between the groups alternatively in every week of trial. DEET, a known standard repellent was used for comparison in this evaluation.

### Ethical clearance

Ethical clearance was reviewed and granted by Ethics committee of Tumaini University at Moshi in Tanzania. Oral and written consents were given to volunteers before starting the study and participation was voluntary.

### Statistical analysis

Total hourly and nightly collections in human landing catch were quantified for *An*. *arabiensis *and *Cx*. *quinquefasciatus*, and biting inhibition was calculated as percentage differences between treated and untreated exposure. The mean mosquito numbers were computed on weekly basis so as to have reasonable number of mosquitoes. The protection efficiency was determined for each week and average for 64 weeks calculated as percentage average protective efficiency following the 4 × 4 Latin square designs. The percentage protection was estimated by Abbot Formula as PE = (N_C _- N_T_)/N_C _× 100%, where N_C _and N_T _are the mean number of mosquito landed on control and on treatment, respectively [18,19]. Data were subjected to analysis of variance (ANOVA) on repeated measures using a Latin square design. Mean percentage protection between treatments and protection against DEET were compared by sample T – tests, and the significance level was determined at P < 0.05.

In the experimental trial, data on mosquitoes found inside, on window traps or verandas, unfed and blood fed for both test and control huts were entered in MS Excel spreadsheet and used to calculate deterrence and feeding inhibition. The results were considered to be significant with an alpha value less than 5%.

## Results

### The efficacy of Ocimum plant extracts in the community field study

In the field study, 1708 *Anopheles gambiae *s.l and 1093 *Culex quinquefasciatus *were collected in 64 weeks of Man Landing Catch. *Anopheles arabiensis *is the commonest Anopheline species accounting for 61.2% of all collected mosquitoes. All *An. gambiae *s.l. were presumed to be *An. arabiensis *following previous identification records in the area [20].

All tested compounds showed significant protection efficiency (PE) to human volunteers against all mosquito species. The PE for *An. arabiensis *was 93.44% for DEET, 91.98% for OS and 89.75% for OK (Table 1). The mean number of *An. arabiensis *caught per night for each treatment and control are shown in Table 1. The lowest and highest numbers of mosquitoes landing per night by type of treatment are indicated as 95% CI.

**Table 1:** Protective efficiency of standard repellent DEET, extracts of *Ocimum suave *and *Ocimum kilimandscharicum *to *Anopheles arabiensis *in the field evaluation estimated by human landing catch conducted for a total of 64 weeks.

Treatment	% Protection	Anopheles collected	Collections/night (Mean ± SE)	95% CI
OS	91.98	137	2.28 ± 0.499	1.29 – 3.28
OK	89.75	175	2.92 ± 0.726	1.46 – 4.38
DEET	93.44	112	1.87 ± 0.474	0.92 – 2.82
Control	24.82	1284	21.4 ± 2.809	15.78 – 27.02

In *Cx. quinquefasciatus*, the PE was 91.30%, 88.65% and 90.50% for DEET, OS and OK respectively. Although the PE of all products was more than 88%, the magnitude of protection by OK and DEET against *Cx quinquefasciatus *was comparable (Table 2). The mean number of *An. arabiensis *caught per night for each treatment and control are shown in Table 2. The lowest and highest numbers of mosquitoes landing per night by type of treatment are indicated as 95% CI.

**Table 2:** Protective efficiency of standard repellent DEET, extracts of *Ocimum suave *and *Ocimum kilimandscharicum *to *Culex quinquefasciatus *in the field evaluation estimated by human landing catch conducted for a total of 64 weeks.

Treatment	% Protection	*Cx. quinquefasciatus *collected	Collections/night (Mean ± SE)	95% CI
OS	88.65	124	2.067 ± 0.695	0.68–3.46
OK	90.5	103	1.72 ± 0.412	0.89 – 2.54
DEET	91.3	95	1.58 ± 0.526	0.53 – 2.63
CONTROL	29.46	771	12.85 ± 2.23	8.37 – 17.32

### The efficacy of Ocimum and other plants in experimental huts

The impact of smoke from burned repellent plant materials in experimental huts was observed in 24 hours after burning. An increase in exophily behaviour (i.e. reduced indoor resting mosquitoes) and blood- feeding inhibition was observed. In experimental huts, high deterrence and feeding inhibition rates of *O. suave*, *O. kilimandscharicum*, *Azadirachta indica, Eucalyptus globules *and *Lantana camara *on *An. arabiensis *and *Cx. quinquefaciatus *were observed by collection of large numbers of these mosquitoes in window and verandah traps. Deterrence ranged from 79.4% to 88.9% and 71.2% to 86.9% while feeding inhibition ranged from 60% to 98.4% and 18.5% to 85.4% in *An*. *arabiensis *and *Cx*.*quinquefaciatus *respectively (Table 3). The OK induce high deterrence in *An. arabiensis *while AI in *Cx. quinquefasciatus *and feeding inhibition (> 90%) in both species of mosquito tested. Performance of OS in terms of deterrence and feeding inhibition of *An. arabiensis *and *Cx. quinquefaciatus *(range from 80% to 98%) was much higher than those of EG and LC (range from 18.5% to 88.1%), in particular LC induced the lowest effect in reducing feeding in Culex (Table 3). In general, the protective effects of the four plant repellents were much higher on *An. arabiensis *than *Cx. quinquefasciatus *mosquitoes.

**Table 3:** Deterrence and feeding inhibition rates of *Ocimum suave*, *Ocimum kilimandscharicum*, *Azadirachta *indica, *Eucalyptus globules *and *Lantana camara *to *An. arabiensis *and *Cx. quinquefaciatus *in experimental huts.

Species	Observed condition	**OS**		**OK**		**AI**		**EG**		**LC**		Deterrence comparison
		Hut A	Hut B	Hut A	Hut B	Hut A	Hut B	Hut A	Hut B	Hut A	Hut B	
	
*An. arabiensis*	Resting Indoors	0	891	04	684	0	784	0	818	39	851	P = 0.07
	Outdoors (WT + VT)	883	101	872	96	902	198	774	92	710	146	
		
	**% Deterrence**	**88.6**		**88.9**		**88.0**		**88.1**		**79.4**		
	
	Unfed	869	171	840	110	879	142	680	104	600	107	
	Fed	14	821	32	670	23	850	94	806	149	890	
	
	**% Feeding inhibition**	**98.4**	**17.2**	**96.3**	**14.1**	**87.9**	**14.2**	**68**	**10.4**	**60**	**10.7**	

*Cx. Quinq*.	Resting Indoors	11	754	0	280	15	843	0	879	94	790	P = 0.10
	Outdoors (WT + VT)	901	179	620	98	922	120	896	102	698	201	
		
	% **Deterrence**	**80.1**		**84.2**		**86.9**		**86.1**		**71.2**		
	
	Unfed	854	189	570	40	833	180	804	91	185	120	
	Fed	47	744	50	215	104	783	92	890	607	871	
	
	**% Feeding inhibition**	**85.4**	**18.9**	**91.9**	**15.7**	**83.3**	**18**	**80.4**	**9.1**	**18.5**	**12**	

Hut A = Experimental hut with slowly burning plant materials and a person.

Hut B = Control Hut with a person alone.

WT = Window trap

VT = Verandah Trap

### The efficacy of Ocimum and other plants in selected community houses

In the selected community houses, feeding inhibition ranged from 61% to 100% for *An*. *arabiensis *and from 50% to 100% for *Cx*. *quinquefasciatus *(Table 4). Likewise, deterrence ranged from 73.1% to 81.9% for *An. arabiensis *and from 56.5% to 67.8% for *Culex*. In particular, the EG induce high deterrence (89.1%) while OS and OK have shown higher feeding inhibition (100%) in both species of mosquito tested. Although the performance of other plants used in terms of (deterrence and) feeding inhibition of *An. arabiensis *and *Cx. quinquefasciatus *(range from 50% to 100%) was higher on treated houses, the difference was comparable to the effect recorded from control houses (range from 55% to 86%) as shown in Table 4. In general, the protective effect of plant repellents was much higher on *Anopheles *than *Culex *mosquitoes.

**Table 4:** Deterrence and feeding inhibition rates of *Ocimum suave*, *Azadirachta indica, Eucalyptus globules *and *Lantana camara *to *An. arabiensis *and *Cx. quinquefaciatus *in village houses.

**Species**	**Character observed**	**OS**		**OK**		**AI**		EG		**LC**		**Deterrence comparisons**
		HS A1	HS B	HS A2	HS B	HS A3	HS B	HS A4	HS B	HS A5	HS B	
	
*An. arabiensis*	Resting indoor	3	63	6	71	2	112	4	85	18	78	P = 0.09
	Outdoors (WT + VT)	94	31	86	22	49	18	59	13	64	21	
		
	**% Deterrence**	**75.2**		**79.6**		**73.1**		**81.9**		**75.3**		
	
	Unfed	3	34	2	35	5	68	3	48	11	43	
	Fed	0	29	0	23	1	44	1	37	7	35	
	
	**% Feeding inhibition**	**100**	**53.9**	**100**	**60.3**	**83.3**	**60.7**	**75**	**56.5**	**61.1**	**55.1**	

Cx. quinq	Resting indoor	2	21	3	33	10	21	2	11	2	7	P = 0.12
	Outdoors (WT + VT)	26	17	31	23	19	11	15	9	19	9	
		
	**% Deterrence**	**56.5**		**57.4**		**63.3**		**62.5**		**67.8**		
	
	Unfed	2	5	2	7	8	6	4	3	2	2	
	Fed	0	16	0	13	2	14	4	8	0	6	
	
	**% Feeding inhibition**	100	23.8	100	35.0	80	30.0	50.0	27.3	100	25.0	

HS A1 to HS A4 = Houses in the village with two occupants in which plant materials were bunt.

HS B1 to HS B4 = Houses in the village with two occupants which were used as Control houses.

**Figure 1:**
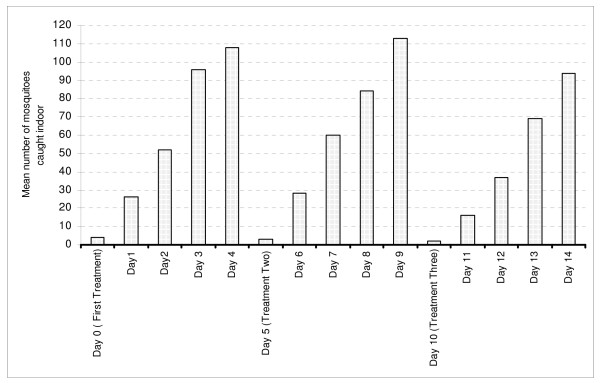
The response of mosquitoes to repellent plant (*Ocimum suave*) burnt in village house in an interval of four days with three rounds of treatment.

However, the deterrence effect of burning repellents in village houses have shown variations between treatment day and days before next treatment in reducing indoor resting mosquito (Figure 1). In general, day 0 of treatment recorded significantly low numbers of mosquitoes resting indoors than subsequent days of observation after treatment. The number of mosquitoes caught increased gradually on subsequent days after treatment, suggesting a lack of residual effect of smoke from repellent plant materials.

## Discussion

The proper use of plant-based repellents against mosquitoes should have a role in reducing insect- borne diseases such as malaria, dengue and filariasis [9]. Most people at risk of malaria infection are in sub- Saharan Africa, the low-income group, disadvantaged people in the community and those living at marginal areas are badly affected by a malaria burden. In communities with low purchasing power, use of plant- based insect repellent is probably the best alternative available [21,22].

### Experimental huts and village house

In both experimental huts and the trial in local village houses, a drastic reduction of indoor resting mosquitoes was observed when plant materials were burnt. The control huts and houses retained high and comparable numbers of indoor resting mosquitoes and blood feeding rates. These showed that the proper (continuous) use of the plant repellents in this area can effectively reduce mosquito population indoor as observed previously in West Africa [5]. Since plant repellents are used in most of modern houses constructed with concrete blocks with wire gauze on the windows this is an indication that, such improvements alone are not enough for protection against biting insects. This observation suggests that the concept of an integrated approach to vector management should be the norm, and therefore making it easier to introduce other control measures [22,23].

Increased knowledge and use of plant based repellent against mosquito bites in the community suggests that local people also see the advantages of plant-derived products over imported synthetic repellents or chemical pesticides [22]. Synthetic chemicals such as organochlorides do not readily degrade in nature and have been identified as ubiquitous pollutants in aquatic ecosystems [24,25]. Concern about the deleterious effects associated with synthetic acaricides and insecticides has revived interest in plants as alternative pesticides for medical, veterinary and crop protection [8]. Even non-human vertebrates use aromatic-arthropod-repellent plants to avoid ectoparasites by rubbing them in the skin [26,27]. Generally, the natural plant products, with a few exceptions, tend to be rather slow-acting, of modest toxicity and rapidly degrade in the environment [28]. Plant- based repellents have multi- active sites in their chemical structures making it difficult for insects to develop resistance. So far, resistance against plant repellents has not been reported [8] as has been the experience with synthetic pyrethroids on bednets [29]. Moreover, plant- based repellents may be more economical than commercially available synthetic chemicals for use in low-income communities. Such cheap, readily available plant repellents can easily be promoted for a wider community use especially in poor rural communities in Tanzania.

### Community trials of plant extracts against biting mosquitoes

In this study, the protective efficacy (PE) of the OS and OK natural products against *An. arabiensis *and *Cx. quinquefaciatus *was high (range: 84% – 89%) and comparable to a standard repellent, DEET (range: 88% to 91%). This agrees with previous studies reporting significantly higher repellence and feeding inhibition against *An. gambiae *and *Cx*. *quinquefasciatus*, and feeding inhibition by OK was higher than OS [9,30,31]. Furthermore, Ocimum species has been reported to reduce the biting activity of the *An. gambiae *by more than 40% in semi-field experiments [32]. The variation in repellence effects between OS and OK ought to be associated with differences in concentrations of other compounds, such as linalool and camphor [30,33]. Linalool is found in high concentrations in OS whereas camphor is found in high concentrations in OK [7]. This variation has been reported to cause different responses in the rate of mortalities in Coleoptera of agricultural importance [34].

Although these natural products have demonstrated a significant protection against malaria and nuisance mosquitoes, their major handicap has been relatively high volatility of many of its monoterpenoid ingredients, leading to rapid loss of protection. In comparison with DEET which has long duration of protection [12] duration of the protective effect by OS and OK extracts was reduced. On the other hand, volatility of ingredients of essential oils contributes to spatial repellence affecting the insects flying in the vicinity [22]. Indeed the formulation to extend the duration of release of volatiles of extracts from repellent plants will improve the protective effects against insects. These data show that natural products emit sufficient quantities of volatiles for protection against mosquitoes under field conditions, as has been reported elsewhere [33,35]. These findings suggest that such natural products can potentially be used as an alternative and cheaper means of malaria control in poor communities.

Among the natural products tested, biting inhibition by OS was generally higher than that of OK. Evidence of higher biting inhibition and protection observed on both OS and OK justify further investigation. This should focus on the improvement of formulations to increase the potency and identify deployment strategies adaptable for local communities.

## Conclusion

This study of two most common plant repellents in north eastern Tanzania, the *Ocimum suave *and *Ocimum kilimandscharicum*, revealed significant protective effect by reducing both the indoor resting mosquitoes and inhibiting mosquito blood- feeding. There is a need to investigate the most efficient deployment methods to the community, product toxicological and safety assessment of extracts or plant fumes, improve formulations and research on the impact of such intervention on disease outcome. The *Ocimum *repellent plants are abundant and locally available, therefore readily acceptable in the community. Moreover, plant- based repellents may be more economical than the commercially available synthetic chemicals for use in low-income communities. Such cheap, readily available plant repellents can easily be promoted for a wider use, especially in poor, rural communities in Tanzania.

## Competing interests

The authors declare that they have no competing interests.

## Authors' contributions

EJK, FWM and EAT designed the study, participated in analysis and interpretation of data and drafted the manuscript. FT, AL and AMM carried out data analysis and interpretation, and were involved in the drafting of the manuscript. EJK, MJM, CM, EL, and MM reared mosquitoes and performed experiments. All authors read and approved the final copy of this manuscript.
